# Mi perro me ha pegado unas verrugas

**DOI:** 10.1016/j.aprim.2021.102069

**Published:** 2021-04-28

**Authors:** Pablo Villagrasa-Boli, Juan Monte-Serrano, Andrea Montes-Torres

**Affiliations:** Servicio de Dermatología y Venereología, Hospital Clínico Universitario Lozano Blesa, Zaragoza, España

El diagnóstico diferencial de las lesiones hiperqueratósicas en la planta del pie, en ocasiones, se reduce a una dicotomía entre las lesiones causadas por el virus del papiloma humano, y los helomas, originados por la presión locorregional mantenida generada por la dinámica de apoyo del paciente.

Sin embargo, existen casos en los que el origen de las mismas puede ser un cuerpo extraño capaz de alojarse en los diferentes estratos dermo-epidérmicos tras perforar la capa córnea, causando así una hiperqueratosis reactiva cuya lesión resultante es semejante al heloma. Esta última entidad, ha sido descrita en la literatura de manera frecuente en las manos o pies de peluqueros y barberos^1^, causada por los microtraumatismos que genera la manipulación del cabello de sus clientes.

En ocasiones, puede presentarse inflamación y sobreinfección local, con formación de abscesos y trayectos fistulosos interdigitales o en las regiones en las que los pelos exógenos se han introducido^2^. Puede llegar a ser tal su semejanza con la enfermedad endógena del folículo piloso, que la literatura se ha llegado a referir a este proceso como sinus pilonidal interdigital^3^. Por este motivo, es importante explorar minuciosamente a los pacientes afectos de hiperqueratosis plantares para tratar de dilucidar cuál es el origen de las mismas, y descartar de este modo que el motivo subyacente de éstas sea un cuerpo extraño. Bajo estas circunstancias, para la resolución y minimización de complicaciones del proceso, es preceptiva la retirada del mismo.

Durante la exploración física, es de gran ayuda la retirada mediante curetaje de la queratina de las lesiones para posteriormente evaluar su imagen dermatoscópica y así poder identificar las estructuras asociadas al papiloma plantar y valorar la posible presencia de cuerpos extraños intralesionales.

En el caso que suscita el presente texto, un varón de 62 años solicitó valoración por presentar desde hace semanas, 3 lesiones plantares dolorosas en el pie izquierdo. Las lesiones habían aparecido poco tiempo después de la adopción de un perro y de su incorporación al domicilio familiar. Inicialmente, fueron diagnosticadas como verrugas virales. Tras realizar una exploración física pormenorizada de las mismas, se pudo observar que se trataba de 3 pápulas hiperqueratósicas amarillentas, de diámetros subcentimétricos variables, en cuyo centro y tras proceder a la retirada de material queratósico, se observaban cuerpos extraños filiformes de color negro (figs. 1A y B). Se procedió a la extracción de los mismos tras curetaje (fig. 1C), y se remitieron a anatomía patológica para identificar su procedencia (fig. 1D). Como tratamiento ambulatorio, se le propuso al paciente el uso de pomadas con ácido salicílico al 50% para eliminar la lesión residual, así como las recomendaciones de no caminar descalzo y el cepillado diario de la mascota.

**Figura 1:**
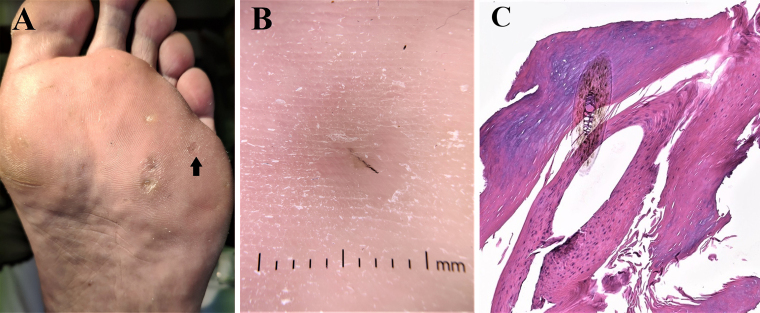
A) Imagen macroscópica de las 3 lesiones por las que consultaba el paciente. B) Examen dermatoscópico en el que se objetiva un cuerpo extraño filiforme atravesando la epidermis (DermLite® DL200 Hybrid). C) Examen anatomopatológico en el que se confirma el origen piloso del cuerpo extraño, con marcada hiperqueratosis reactiva perilesional.

La no correcta filiación de este tipo de procesos, puede ser la causa de evolución tórpida de las lesiones, de la aparición de nuevas, y de la instauración de múltiples tratamientos queratolíticos o físicos, que pueden ser causa de iatrogenia y disminución de la calidad de vida de estos pacientes.

## Financiación

Para la elaboración del presente artículo no se ha recibido ningún tipo de ayuda ni financiación económica por parte de entidades públicas o privadas.
